# Autogenic Training Improves the Subjective Perception of Physical and Psychological Health and of Interpersonal Relational Abilities: An Electronic Field Survey During the COVID-19 Crisis in Spain

**DOI:** 10.3389/fpsyg.2021.616426

**Published:** 2021-07-27

**Authors:** Luis de Rivera, Naiara Ozamiz-Etxebarria, María Dosil-Santamaría, Leonor de Rivera-Monterrey

**Affiliations:** ^1^Institute of Psychotherapy and Psychosomatic Research, Madrid, Spain; ^2^Department of Developmental and Educational Psychology, University of the Basque Country UPV/EHU, Leioa, Spain; ^3^Faculty of Education, University of the Basque Country, Leioa, Spain; ^4^Department of Research and Diagnostic Methods in Education, University of the Basque Country UPV/EHU, Leioa, Spain

**Keywords:** COVID-19, autogenic therapy, anxiety, autogenics, empathy

## Abstract

Currently, humanity is facing one of the most critical situations of this century, the COVID-19. The adverse effects of the pandemic on the mental health of the population are well known. Fear of illness, confinement, lack of financial resources, or poor social support can influence people’s mental state. Despite these risks, several psychological resources may help address this situation. The present study investigated the effectiveness of a self-relaxation techniques known as autogenic training. Autogenic training is a well-known method in Europe for the treatment of anxiety and stress disorders. The practice of autogenic training is also reported to improve neurovegetative and immune regulation. This study focuses on describing how autogenic training is helping its practitioners to cope with the pandemic. Specifically, they report strong beneficial effects on their physical, psychological, and relational health. In total, 75 autogenic training practitioners (41 women), age 22–71, participated in the survey. An *ad-hoc* questionnaire was developed to collect information on sociodemographic variables, health status during the pandemic, characteristics of their AT practice, and response to the pandemic as outcome variables. The questionnaire was distributed through Google Forms in the first week of September 2020. The results show that there was an increase in the practice of autogenic therapy during the pandemic, especially among women. In addition, the majority of participants (88%) remained healthy during the pandemic. Furthermore, the results show that autogenic training is very useful for physical and psychological health and for a better understanding of others. Therefore, the practice of autogenic training is recommended to people who live moments of anxiety, are afraid of illness, or feel that they have to improve the quality of relationships with others.

## Introduction

Humanity is now facing an unprecedented crisis with medical, psychological, economic and social aspects. The COVID-19 December 2019 outbreak in Wuhan spread rapidly to all the world (Chen et al., 2020) and by March 2020 made its full impact on Spain. Exceptional measures were taken, including the confinement of all large sectors of the population (Arango, 2020; Ozamiz-Etxebarria et al., 2020b). Severe negative effects in mental health have been reported, mainly related to fear of contagion (Ammar et al., 2020b,c; Shigemura et al., 2020) and adaptation to the confinement (Qiu et al., 2020). Psychological resources have been applied to ease this psychological impact (de Rivera, 2020; Lupe et al., 2020; Ozamiz-Etxebarria et al., 2020a), including computer-assisted distance training in relaxation (Wei et al., 2020).

In a previous experimental study, we reported the efficacy of relaxation techniques, including autogenic training, on easing COVID-19-related anxiety on young university students in the Basque Country (Ozamiz-Etxebarria et al., 2020a). Autogenic training (AT), created by Johannes Heinrich Schultz (1932) as a method of “concentrative self-relaxation,” promptly became a standard tool in European psychosomatic medicine and clinical psychology (Hoffmann, 2017). Sustained regular practice decreases stress reactivity (Henry et al., 1991), enhances emotional stability (Carruthers, 1979), reduces trait anxiety, and increases the sense of personal control (Farnè and Jimenez-Muñoz, 2000). Initially, regarded as a variant of auto-hypnosis, autogenics is now more appropriately considered a non-Buddhist meditation/mindfulness method, able to facilitate personal development and to increase resilience to stress and sturdiness in emotional crisis (Carruthers, 1979; de Rivera, 2018).

The present survey focuses on the beneficial effects of the practice of AT during the pandemic in 75 usual practitioners of autogenic training (hereafter AT) living in Spain. Specifically, we inquired about their health status during the pandemic, their COVID-19-related anxiety, and how AT practice is helping them to maintain their physical, psychological, and relational health.

We anticipated that demographic variables, such as age, gender, and geographical location (see Section “Participants”), may have a relation with our outcome variables. Other variables that we anticipated having an effect on our outcome variables are the frequency of AT practice and the AT seniority (number of months practicing AT). The outcome variables are COVID-19-related anxiety and the evaluation by the participants of the degree in which AT practice helps in their psychological, physical, and relational health.

## Materials and Methods

### Participants

The participants were 75 practitioners of AT (58.7% from Madrid; 41.3% from other regions of Spain); 41 were women (54.7%), and 34 were men (45.3%). Their mean age was 50.92 years (SD = 11.19) with a range of 22 to 71 years. As for their professions, 33 (44%) of them were health professionals (medical doctors 18.7%, psychologists 17.3%, and other healthcare workers 8%), 27(36%) were other professionals, 12(16%) were clerical and services workers, and 3(4%) were retired. The inclusion criterion was that they were trained in AT and practiced the method regularly. The exclusion criterion was that they were not of legal age.

### Measures and Instruments

An *ad-hoc* questionnaire was developed to collect information on sociodemographic variables, characteristics of AT practice, health status, and participants’ opinion on the benefits of their AT practice.

The variables studied are described below:

*The sociodemographic variables* studied were gender, age, place of residence, and profession.

*The characteristics of AT practice* were the frequency of practice at normal times, the frequency during the pandemic, and the length of time they had been practicing the AT method.

Specifically, the questions were as follows:

- For how long have you been practicing autogenics? Please indicate years, months and days.
- How many times a week do you usually practice autogenic training?
- Since the pandemic began to spread, how many times a week have you practiced autogenic training?

*The health status variable* asked was whether they had become ill since the pandemic began, and if so, from what illness.

The specific question was as follows:

- Have you become ill since the pandemic began? If so, please indicate the illness.

*The questions on the benefits of AT practice* were (responses were graduated on a Likert scale from 0 to 10):

- Do you think autogenic training has helped you to stay psychologically healthy?
- Do you think autogenic training has helped you to stay physically healthy?
- Do you think autogenic training has helped you to understand others better?

Finally, *the question on anxiety created by the COVID-19 pandemic was as follows*:

- Are you anxious about being infected by the COVID-19 virus?

### Procedure

One hundred graduates from the Madrid International Committee of Autogenic Therapy (ICAT) Centre were contacted by email through the ICAT databases and asked to complete an online questionnaire on their demographic data, details of their autogenic practice, and their response to the pandemic. The study had the approval of the Ethics Committee for Research Related to Human Beings of the University of the Basque Country. All subjects participated on a voluntary basis, received information about the procedure of the investigation, and gave their informed consent. Therefore, the procedure complied with the requirements of the Helsinki Declaration of the World Medical Association.

The *ad-hoc* questionnaire was distributed through Google Forms in the first week of September 2020. After analyzing the database in Microsoft Excel,^1^ 25 questionnaires showing a pattern of non-response in certain items were removed from the total sample. The study fulfilled all the provisions of Law 15/1999 on the Protection of Personal Data and, in addition, participants gave their informed consent on a voluntary basis before completing the questionnaire.

### Data Analysis

The data extracted from the Google Forms questionnaire were imported into the IBM SPSS v.26 (SPSS Inc., Chicago, IL) for Windows for descriptive and inferential analyses of the sample.

Through the Kolmogorov–Smirnov test, we found that all variables, except age, followed a non-normal distribution, so we performed non-parametric tests in the inferential analysis. We used the non-parametric Mann–Whitney *U* test to compare the differences in the AT variables and in the outcome variables by gender, profession, and region of residency. We carried out the Spearman’s correlation coefficient to ascertain the correlation between age and AT variables and outcome variables, and also to ascertain correlations between the different AT variables and outcome variables. We used the Wilcoxon signed-rank test to compare the usual frequency of AT practice and the practice during the pandemic.

## Results

### AT Variables

The usual frequency of AT practice (number of exercises per week) for the overall sample is 8.64 (*SD* = 6.48, range from 1 to 21) and by gender is 7.27 (*SD* = 5.81) for women and 10.29 (*SD* = 6.92) for men.

The frequency of AT practice increased during the pandemic to 10.01 (*SD* = 7.04, range from 1 to 31) exercises per week for the overall sample. By gender, women practice increased to 8.66 (*SD* = 6.84) exercises per week and men to 11.65 (*SD* = 7) exercises per week. The increase in AT frequency of practice during the pandemic is significant in the general sample (*Z* = −3.05, *p* < 0.002).

AT seniority has a median of 18 months and a mean of 63 months (*SD* = 115.20, range from 2.5 months to 480 months). There are significant differences by profession, with healthcare workers having greater AT seniority than other professions (*M* = 112.93, *SD* = 152.10 vs. *M* = 23.75, *SD* = 48.11; *U* = 265.00, *p* < 0.001). There is also a positive correlation between age and AT seniority, *r* = 0.313 (*p* < 0.006).

### Health Status

Nine persons of the sample became ill during the pandemic (12%) and 66 remained in good health (88%). Of those who became ill, four were infected by the COVID-19 virus (5.3%), three reported psychological disturbances (4%), and two other medical disorders (2.7%; Table 1).

**Table 1:** AT patterns of practice and AT seniority by health status.

Health status	*n*	*Fr*	Usual practice	Pandemic practice	AT seniority
Healthy	66	88	8.45 (6.18)	9.69 (6.92)	67.27 (121.74)
COVID-19	4	5.3	8.50 (8.58)	9.75 (7.54)	54.00 (39.80)
Psychological	3	4	4.67 (4.73)	10.33 (9.05)	20.00 (6.93)
Medical disorders	2	2.7	21.00 (0.00)	21.00 (0.00)	4.35 (0.92)

Those infected by the COVID-19 virus were three women and one man; their mean age was 47 years with a range of 37 to 60 years. One of them was asymptomatic, and the other three had a mild course at home with no need for hospitalization. All of them were from Madrid, the region of Spain most affected by the COVID-19 virus, with an infection rate of 13.3% at the time of the study, 7.9% being the general prevalence in Spain (Metroscopia, 2020).

All the participants maintained or increased their AT practice during the pandemic, although statistical significance for the increase of practice was reached only in the group of those who remained healthy (increase *M* = 1.23, *SD* = 4.42; *Z* = −2.630; *p* < 0.009).

### Outcomes: AT Helps Psychologically, Physically, and in Understanding Others, and COVID-19-Related Anxiety

Table 2 reflects the assessment of AT benefits (psychological, physical, and relational) and COVID-19-related anxiety by health status. As for the participants’ evaluation of the degree AT practice has helped them to keep psychologically healthy, on a Likert scale from 0 to 10, the ratings for the general sample were 8.03; (*SD* = 1.81). By gender, women rated 8.36 (*SD* = 1.31) and men, 7.52 (*SD* = 2.33); by profession, healthcare workers 8.04 (*SD* = 1.61) and other professions 8.03 (*SD* = 1.98); and by site of residence, those living in Madrid rated 7.69 (*SD* = 1.66) and those from other regions of Spain 8.44 (*SD* = 1.93).

**Table 2:** Health status, evaluation of AT benefits (psychological, physical, and relational) and COVID-19-related anxiety.

Health status	AT helps psychologically	AT helps physically	AT helps understand	COVID-19 anxiety
Healthy	8.02 (1.80)	7.34 (1.80)	7.38 (2.22)	4.25 (2.64)
COVID-19	8.25 (1.36)	7.50 (1.73)	8.50 (1.00)	3.50 (2.52)
Psychological disturbances	8.00 (3.46)	8.33 (2.89)	8.33 (2.89)	7.33 (2.52)
Medical disorders	8.00 (0.00)	7.00 (0.00)	9.00 (1.41)	7.00 (0.00)

As to what extent AT practice has helped them to stay physically healthy, the overall sample rated 7.39 (*SD* = 1.81). By gender, women, 7.53 (*SD* = 1.61) and men, 7.20 (*SD* = 2.08); by profession, healthcare workers 7.24 (*SD* = 2.19) and other professions 7.50 (*SD* = 1.52); and by site of residence, those from Madrid rated 7.00 (*SD* = 1.81) and those from other regions 7.89 (*SD* = 1.72).

As to what extent AT practice has helped them to better understand others, the overall ratings are 7.57 (*SD* = 2.17). By gender, women rated 7.68 (*SD* = 2.07) and men 7.46 (*SD* = 2.30); by profession, healthcare workers 7.77 (*SD* = 1.86) and other professionals 7.40 (*SD* = 2.43); and by site of residence, those from Madrid rated 7.41 (*SD* = 1.88) and those from other regions 7.79 (*SD* = 2.54).

There are significant differences only to the degree in which AT helps psychologically by site of residence, which is slightly lower in Madrid than in the other regions of Spain (Madrid *n* = 44, *M* = 8, *SD* = 1.52; other regions of Spain, *n* = 31, *M* = 8.52, *SD* = 1.81; *U* = 302.00, *p* < 0.034).

As for the COVID-19-related anxiety, in a rating from 0 to 10, the total sample mean was 4.38 (*SD* = 2.68). By gender, women rated 3.95 (*SD* = 2.65) and men 4.88 (*SD* = 2.66); by profession, healthcare workers, 4.27 (*SD* = 2.44) and other professions 4.46 (*SD* = 2.89); and by site of residence, Madrid 4.24 (*SD* = 2.67) and other regions 4.55 (*SD* = 2.72). There is a non-significant and negative correlation (*r* = −0.041) between age- and COVID-19-related anxiety (*p* < 0.732).

As shown in Table 3, there are significant positive correlations between the usual frequency of AT practice and the evaluation of the degree in which AT helps psychologically (*r* = 0.321), physically (*r* = 0.266) and in understanding others (*r* = 0.433). Regarding the frequency of AT practice during the pandemic, there are significant positive correlations only with the evaluation of the degree in which AT helps understand others better (*r* = 0.291). Therefore, the more frequent the usual AT practice, the higher the evaluation of its psychological, physical, and relational benefits, whereas the frequency of AT practice during the pandemic only correlates with the ability to understand others.

**Table 3:** Spearman’s correlation coefficient between AT variables and outcomes.

S. No.		1	2	3	4	5	6
1.	Usual frequency						
2.	Pandemic frequency	0.852^**^	–				
3.	AT seniority	−0.098	0.148	–			
4.	AT psychologically	0.321^*^	0.209	−0.031	–		
5.	AT physically	0.266^*^	0.188	−0.028	0.822^**^	–	
6.	AT empathy	0.433^**^	0.291^*^	0.050	0.818^**^	0.821^**^	–
7.	Afraid of COVID-19	0.042	−0.037	−0.013	−0.021	−0.003	−0.162

*n = 75.*

* *p < 0.05*;

** *p < 0.001*.

## Discussion

The descriptive data of the study show a similar proportion of women (54.7%) to men (45.3%). Nearly half of them were healthcare professionals, and almost half of the sample resided in the city of Madrid. The usual frequency of AT practice, measured by the number of AT exercises per week, is 8.64 times/week on average. The recommended frequency ranges from 7 to 21 times per week, being somewhat higher during the learning period of the technique (3 times/day), whereas experienced trainees tend to practice once a day (7 times/week). As for gender differences, men tend to practice AT more times per week than women, both during normal times and during the pandemic. In addition, both women and men increased their AT practice during the pandemic, women to a greater extent.

The increase of practice during the pandemic may reflect a response to higher levels of stress and anxiety, which is concordant with the findings of Ozamiz-Etxebarria et al. (2020a) on the effects of AT on easing COVID-related anxiety in an experimental study with university students. This increase is more significant in women probably because they tend to experience higher anxiety during the pandemic (Lai et al., 2020; Liu et al., 2020).

The positive correlation between age and AT seniority may seem obvious, as older people have had more years to practice than younger ones; but it may also indicate that AT practice, once initiated, tends to become a life-long habit. The somewhat counterintuitive negative correlation found between COVID-related anxiety and age corroborates other studies showing that older people present less anxiety than younger ones during the current pandemic (Picaza Gorrochategi et al., 2020).

In terms of professions, the most significant finding is that health professionals have been practicing longer than other professionals. This is probably because health professionals have had easier access to AT and therefore may have started practicing earlier than others.

Fear of contagion has been one of the most frequent psychological reactions in the population during the current pandemic. However, the fear levels are low in our overall sample, as on a scale from 1 to 10 women rated an average of 4.20 and men an average of 5. The tendency of men to show more COVID-19-related anxiety than women is in contradistinction to a study in Cuba reporting significantly greater fear of COVID-19 in women (Broche-Pérez et al., 2020) and to several other studies showing that women have had more anxiety during the pandemic (Lai et al., 2020; Liu et al., 2020). The lower anxiety scores in women in our sample may be explained by their greater increase of AT practice during the pandemic. These low levels of COVID-related anxiety in our sample may be related to the practice of autogenic training, and effect already shown in an experimental study by Ozamiz-Etxebarria et al. (2020a).

It is interesting that most participants (88%) remained healthy during the pandemic, and only 5.3%, all from the Madrid region, became infected by the COVID-19 virus. Taking into account that the calculated prevalence of infection at the time of the study was 13.3% for the Madrid region (Metroscopia, 2020), the results of the present sample are quite optimistic and may suggest that AT may have a protective effect against virus infection.

We found that participants who remained healthy increased significantly their AT practice during the pandemic, which may indicate that increased practice has both physical and psychological health benefits.

Participants who were infected by the COVID-19 virus had the least COVID-related anxiety whereas those who became ill for other causes had the greatest. This may seem obvious, as those who had already experienced the COVID-19 infection may have felt relieved by the mild course of the illness and also protected against repeated infection, whereas those ill for other causes may have increased fear of their condition being complicated by COVID-19.

Another positive fact of the present study, which points to the benefits of AT, is that the people who have remained healthy throughout the pandemic, without contracting any type of disease, are precisely those who have been practicing AT for more years, with an AT seniority about twice those who became ill. This finding confirms previous data showing that, besides the immediate effects of the AT exercises on neurovegetative function, regular practice over a number of years permanently increases the body’s homeostatic functions and thus increases resilience to illness (Luthe and Schultz, 1970a). More recent research has shown that AT improves the immune function (Minowa and Koitabashi, 2014).

According to the participants, AT has a positive effect on their psychological and physical health and in their ability to understand others. This perception tends to be somewhat higher in women, although it does not reach statistical significance. Nor are there any statistically significant differences when comparing the psychological, physical, and relational effects in terms of professions. This leaves the question open for further studies with a larger number of participants. In terms of site of residence, it seems that people living in other regions of Spain perceive that AT is helping them more than those living in Madrid. This occurs at both psychological and physical levels, as well as in understanding others. Madrid is one of the hardest hit cities, not only by COVID-19 but also by constant changes in confinement regulations (Mucientes, 2020), which may create discouragement among its population. However, although the difference is not significant, it seems that they also tend to have less anxiety about COVID-19. Perhaps, the fact of observing so much contagion in the city diminishes the fear because the virus is better known to the people of Madrid.

Participants value AT highly as helpful in maintaining psychological health. The degree of positive evaluation of the benefits of AT correlates with the usual frequency of AT practice. This is a well-known effect of AT, and achieving emotional stability is one of the main reasons for its application in clinical psychology (Luthe and Schultz, 1970b; Hoffmann, 2017). Autogenic training has also been reported to improve psychological wellbeing in chronic medical patients (Ramirez-Garcia et al., 2020).

Participants also value AT as highly helpful in maintaining physical health. As previously, this perception correlates with the usual frequency of practice and is concordant with research showing that AT practice increases the body’s homeostatic functions and the resilience to illness (Luthe and Schultz, 1970a). Furthermore, a meta-analysis of clinical outcome studies has shown positive effects of AT for tension headache, migraine, mild-to-moderate essential hypertension, coronary heart disease, bronchial asthma, unspecified somatoform pain disorder, Raynaud’s disease, anxiety disorders, dysthymia, mild-to-moderate depression, and functional sleep disorders (Stetter and Kupper, 2002).

In terms of relating better to others, the average scores are also high, showing that AT may help to overcome the relationship difficulties associated with confinement (Brooks et al., 2020). The practice of AT is known to increase empathy and other positive personality traits, an effect called “the third autogenic switch” (de Rivera, 2018; Ross, 2020). As expected, this third autogenic switch is correlated in our sample with the frequency of practice, both in usual times and during the pandemic.

Social relationships have also been damaged during this pandemic, as strong measures of social distancing and confinement have totally changed the way people relate to each other. Isolation and loneliness have been the major relational problems of this pandemic (Brooks et al., 2020; Smith and Lim, 2020). Therefore, the perception that AT helps to understand others better is a further argument in favor of its practice.

AT seniority does not significantly influence the evaluations of the effects of AT on physical, psychological, and relational health, which may indicate that people who have been practicing AT for a short time are as aware of its benefits as those who have been practicing over longer periods. In other words, it seems that the positive effects of autogenic training are noticeable from the very beginning of its practice.

The strength of this research is that it is the first study on the benefits of autogenic training in coping with the adverse psychological effects of the pandemic. The two main limitations of the study are the small size of the sample, which may prevent several tendencies to reach statistical significance, and the failure of taking into account in the selection criteria the health status of the participants, so to exclude its influence in our results. These aspects should be taken into account in the future studies.

## Conclusion

Practitioners of autogenic training consider this practice highly helpful to their physical and psychological health and for the better understanding of others. These three aspects are very important during the COVID-19 pandemic. Social relations have deteriorated due to social isolation (Ammar et al., 2020a; Hickie, 2020), and AT is useful in facilitating empathy. Psychological health has suffered during the pandemic, as shown in different studies (Ozamiz-Etxebarria et al., 2020b), and the physical dimension is obvious in times when the risk and fear of getting sick, especially of COVID-19, have increased significantly (Ornell et al., 2020). Therefore, we recommend the practice of autogenic training to people who are living moments of anxiety, are afraid of the disease or feel they have lost the quality of relationships with others.

## Data Availability Statement

The original contributions presented in the study are included in the article/supplementary material, further inquiries can be directed to the corresponding author.

## Ethics Statement

The studies involving human participants were reviewed and approved by the Ethics Committee for Research Involving Human Subjects. The patients/participants provided their written informed consent to participate in this study.

## Author Contributions

LR, LR-M, and NO-E were involved in the conceptualization of the project and in the acquisition and analysis of the data. MD-S was involved in the interpretation of the data. All authors were involved in the drafting and revising of the work for intellectual content, provided approval for submission of the contents for publication, and agreed to be accountable for the accuracy and integrity of the project.

## Conflict of Interest

The authors declare that the research was conducted in the absence of any commercial or financial relationships that could be construed as a potential conflict of interest.

## Publisher’s Note

All claims expressed in this article are solely those of the authors and do not necessarily represent those of their affiliated organizations, or those of the publisher, the editors and the reviewers. Any product that may be evaluated in this article, or claim that may be made by its manufacturer, is not guaranteed or endorsed by the publisher.

## Footnotes

^1^ https://products.office.com/
